# Syndrome of inappropriate antidiuretic hormone secretion after functional endoscopic sinus surgery

**DOI:** 10.1093/jscr/rjab603

**Published:** 2022-01-20

**Authors:** Cezar Octavian Morosanu, Keng Siang Lee, Fatemeh Keshtkar, Claire Langton-Hewer

## Abstract

Functional endoscopic sinus surgery (FESS) is effective in cases of sinusitis where pharmacological treatment has not been successful. Patients undergoing FESS have reported an 85% improvement in symptoms as measured by the quality of life scores. Despite its convincing therapeutic benefit, complications sometimes occur with potentially dire consequences. We report the case of a 69-year-old patient who underwent FESS for recurrent frontal sinusitis and developed a syndrome of inappropriate antidiuretic hormone secretion (SIADH) on Day 3 post-operatively. To our knowledge, this is the first documented case of SIADH arising after an endoscopic intervention for frontal sinusitis.

## INTRODUCTION

Functional endoscopic sinus surgery (FESS) is used to treat sinusitis where pharmacological treatment has not been successful. Despite being perceived as a relatively safe procedure, FESS has been reported to have a complication rate varying from 0.36 to 3.1% [1, 2]. Frontal sinusitis can have severe complications [3], although surgical treatment is not without its own risk. Here, we describe syndrome of inappropriate antidiuretic hormone secretion (SIADH) resulting from an endoscopic intervention for frontal sinusitis.

## CASE PRESENTATION

A 69-year-old female patient presented with a significant headache after undergoing a FESS intervention 3 days prior to admission. She had a previous medical history of Type 2 diabetes mellitus, gastric reflux, epiphora, allergic rhinitis and chronic sinusitis for which she had undergone multiple FESS procedures. Her regular medication included insulin, saxagliptin, metformin, fexofenadine, ezetimibe, fluvastatin and lansoprazole and she was allergic to tetracycline antibiotics. The patient was well after the surgery. Her pain was adequately managed with paracetamol and codeine and she was discharged the next day. However, her minor headache worsened over the following days. She subsequently developed nausea, vomiting and photophobia, which eventually led the patient to present to the emergency department with suspected meningitis.

## INVESTIGATIONS

**Figure 1:**
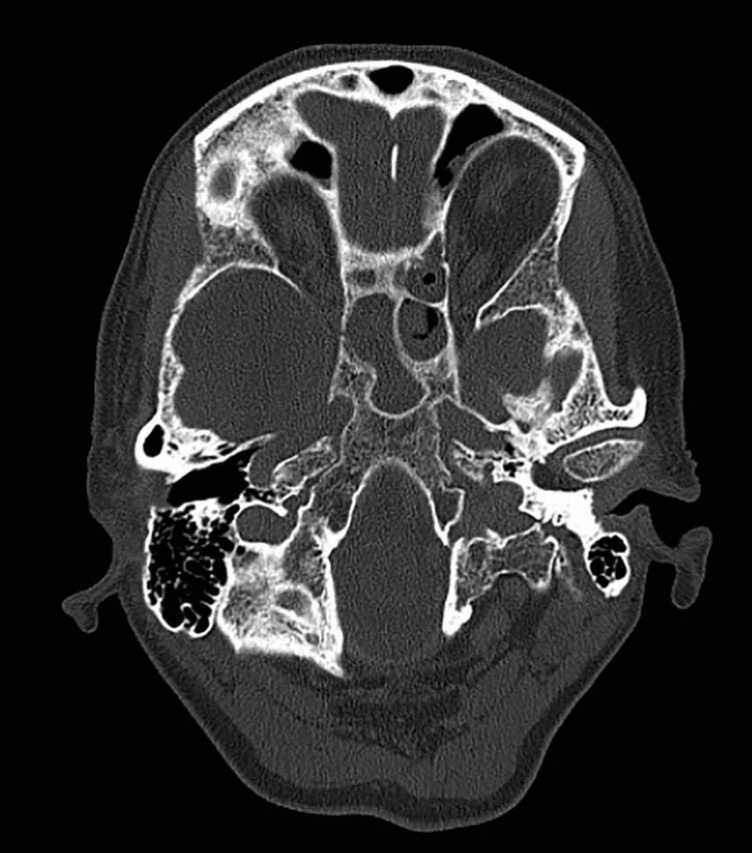
CT head revealed no signs of meningeal or intracerebral enhancement to suggest an intracranial abscess or cerebritis, but it suggested significant opacification in the paranasal sinuses, particularly in the ethmoid and sphenoid sinuses, most likely indicating a minor post-operative haemorrhage.

Computed tomography (CT) of the head revealed no signs of meningeal or intracerebral enhancement to suggest an intracranial abscess or cerebritis (Fig. 1).

Blood tests were also performed and a significant hyponatraemia (122 mmol/l, and normokalaemia: 4.2 mmol/l) was incidentally diagnosed. Physical examination revealed no signs of dehydration or fluid overload (skin turgour was normal, and no pitting oedema was identified), pointing to a clinical diagnosis of dilutional hyponatraemia.

Relevant markers of fluid status such as haematocrit (0.386 l/l), albumin (35 g/l), urea (2.5 mmol/l) and creatinine (42 μmol/l) were within reference ranges, suggesting dilutional hyponatraemia. Given this information, further plasma and urine osmolality testing of 257 and 627 mOsmol/kg, respectively, and urine sodium of 87 mmol/l revealed the diagnosis of SIADH, and appropriate fluid restriction was commenced. She was later transferred to an endocrinology ward where she fully recovered after tightening fluid restriction and stopping her lansoprazole treatment.

A chest X-ray was performed to exclude the possibility of ectopic ADH secretion from a small cell carcinoma. Renal, adrenal and thyroid function tests were performed to rule out any other cause for the patient’s presentation, however, all results were within normal limits. A short Synacthen test raised serum cortisol from 55 to 804 nmol/l, which revealed normal adrenal function and excluded adrenocorticotrophic hormone deficiency. As the patient had mild abdominal discomfort, upper gastrointestinal endoscopy and ultrasound (US) abdomen were performed, which yielded no significant findings.

The severe hyponatraemia, decreased plasma osmolality and inappropriately concentrated urine with continued natriuresis (urine sodium of 87 mmol/l), compounded by clinical evidence of euvolaemia and normal adrenal, renal function, led to the final diagnosis of SIADH.

## DIFFERENTIAL DIAGNOSIS

SIADH is essentially a diagnosis of exclusion and cannot be diagnosed on insufficient evidence without regard to other possible causes of hyponatraemia. In many cases, the cause of hyponatraemia can be recognised clinically by the volume status of the patient—physical examination of the patient revealed no signs of dehydration or fluid overload (skin turgour and mucus membranes were normal, and no pitting oedema was seen), pointing the clinical diagnosis to a dilutional hyponatraemia. Effective serum osmolalities revealed a hypotonic hyponatraemia [4].

Clinically, differentials of hyponatraemia such as cerebral salt wasting (CSW), Addison disease, renal failure, hypothyroidism, chronic heart failure, gastrointestinal abnormalities and cirrhosis [4] could be excluded by our patient’s clinical findings of euvolaemia, blood tests and medical history. It is possible to differentiate clinically between CSW and SIADH: patients with CSW display hyponatraemia in association with the clinical and biochemical features of hypovolaemia with marked natriuresis and diuresis. Patients with SIADH display clinical features of euvolaemia and biochemical findings of dilutional hyponatraemia. This distinction is vital because the management of the two conditions is significantly different.

Once the diagnosis of SIADH was established, the main goal was to investigate causes that could have generated the electrolyte imbalance. Any central nervous system disorder, such as meningoencephalitis, cerebral tumours, cavernous sinus thrombosis, hydrocephalus, multiple sclerosis, subarachnoid haemorrhage or head trauma, was excluded by the CT head scan. Pulmonary conditions, such as bronchogenic small cell carcinoma, pneumonia, tuberculosis, abscess and aspergilloses, were considered but were ruled out by a clear chest X-ray. As the patient had mild abdominal discomfort, upper gastrointestinal endoscopy and abdominal US were performed, and we excluded any gastrointestinal carcinoma. Potential endocrine issues, such as adrenal insufficiency and hypothyroidism, were part of the differential diagnosis but were excluded after a positive short Synacthen and findings of normokalaemia and normal thyroid function tests. The patient’s regular medication did not have any drugs that stimulate ADH release of potentiate its actions, or ADH analogues [4]. However, the small dose lansoprazole she was taking for her gastroesophageal reflux may have contributed to her hyponatraemia. Vomiting is a symptom of severe hyponatraemia and it is likely that these episodes also had a role in further aggravating her sodium levels through dehydration. Another potential contributing factor could have been the analgesic medication given post-operatively. Hereditary causes were excluded from her medical history. She had no history of pituitary surgery, but the small haemorrhage in her sphenoid sinus, visible on the CT imaging, could have caused an imbalance in the function of her neurohypophysis.

## TREATMENT, OUTCOME AND FOLLOW-UP

The patient was put on a strict fluid restriction of 1500 ml/day for 3 days. No improvement was noted with this regimen, so the restriction was tightened to 750 ml/day for an additional 6 days. In addition, lansoprazole was also stopped on Day 7 as it may have potentiated the hyponatraemia. Over the next week, her hyponatraemia steadily and significantly improved and the patient was safely discharged. All electrolytes were in range on her follow-up 16 days post-FESS (Table 1). No further follow-up was required.

**Table 1:** Investigation results

Investigation	Post-FESS day 1	Post-FESS day 2	Post-FESS day 3	Post-FESS day 4	Post-FESS day 5	Post-FESS day 6	Post-FESS day 7	Post-FESS day 8	Post-FESS day 9	Post-FESS day 16
Serum sodium (mmol/l); normal range: 133–146	122	123	122	118	121	123	128	133	135	137
Serum potassium (mmol/l); normal range: 3.5–5.3	4.2	4.8	4.2	4.1	4.2	4.2	4.5	4.2	4.7	4.7
Urea (mmol/); normal range: 2.5–7.8	2.5	3.9	4.9	3.0	2.8	3.9	3.4	3.3	3.4	3.4
Creatinine (μmol/l); normal range: 45–84	42	55	47	46	57	57	55	62	55	56

## DISCUSSION

Endocrine disorders are a rare consequence of sinus pathology and are even rarer post-sinus surgery [5]. Another case of SIADH has also been reported in a 33-year-old male patient who underwent sinus surgery for a sphenoid sinusitis [6]. The cause for this clinical entity was assumed to be the surgical stress to the pituitary gland due to the perforation of the base of the sellar region [6]. Different reports describe SIADH as the initial presentation of isolated esthesioneuroblastomas in the maxillary sinus [7–10].

In our case, the endoscopic surgery to the frontal sinus triggered an endocrine imbalance almost immediately after surgery. It was considered that SIADH originated in the insult to the pituitary gland as a consequence of surgical stress, similar to the case by Takeda *et al*.; however, there are clear indications that factors, such as the lansoprazole treatment and her episodes of vomiting, could have contributed to her low sodium [6]. SIADH is a known complication of endoscopic transnasal, transsphenoidal interventions for pituitary masses due to the above mechanism [11]. An indication for this process in our case was the small degree of bleeding evident in the sphenoid sinus. Equally, it should be emphasized that there is a high possibility that the sinus surgery was just a contributing factor in this patient and that SIADH was in fact caused by the lansoprazole treatment [12]. Interestingly, Falhammar *et al*. revealed that there is no association between hyponatraemia and the newly started and ongoing lansoprazole treatment [13].

## CONCLUSIONS

We report an unusual case of severe hyponatraemia associated with FESS, although the exact pathophysiology of SIADH induced in our patient remains unclear. Nevertheless, it is important for physicians to investigate the underlying cause of refractory or recurrent hyponatraemia, as it can be a marker of underlying disease.

## CONFLICT OF INTEREST STATEMENT

None declared.

## Funding

Funding to pay the Open Access publication charges for this article was provided by the University of Bristol (to Keng Siang Lee).
